# Supplementary material for: Extremely large magnetoresistance in twisted intertwined graphene spirals
Source: Nat Commun. 2024 Jul 20;15:6120. doi: 10.1038/s41467-024-50456-0 (PMC11271300; doi:10.1038/s41467-024-50456-0)
Supplement: Supplementary file 1 — Supplementary Information [file 41467_2024_50456_MOESM1_ESM.pdf]

Supplementary Information for

Extremely large magnetoresistance in twisted intertwined graphene spirals

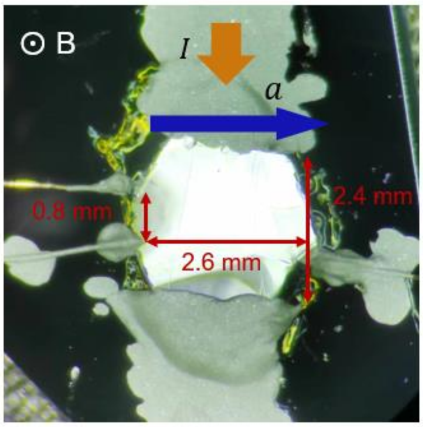

Supplementary Figure 1 The experimental setup employed for resistivity measurement. Our crystal, featuring a single spiral, exhibits a thickness of approximately 70 μm. Notably, naturally formed edges are discernible, with one specifically identified as the a-axis. The application of current density is perpendicular to the a-axis, while the magnetic field aligns parallel to the c-axis, designated as the direction of spiral growth. Electrodes, derived from silver glue, span both sides and surfaces of the sample. The distance between the two vertical voltage terminals measures 0.8 mm, while the separation between the two horizontal terminals is 2.6 mm.

Angular-dependent magnetoresistance.

To further confirm the certain twist angle in long range, we also studied the angular-dependent in-plane magnetoresistivity. During the in-plane rotation of the magnetic field, the current was applied along in-plane while the initial field direction ($\Phi = 0°$) is set to be parallel to current as shown in schematic image in Supplementary Figure 2a. The angle-dependent resistance R at 2 K and 9 T reveals the symmetry of the orbital part of the electronics structure which corresponds to the crystal lattice. It is expected that a two-fold symmetry for the in-plane resistance would occur due to the Lorentz force since the current were applied within the *ab*-plane as well (Supplementary Figure 2b). Nevertheless, besides the background of the two-fold symmetry, there are additional oscillations caused by other contribution. Thus, we subtract the background from the two-fold symmetry component, and observe other periodic signals as can be visualized in the middle and bottom of Supplementary Figure 2b. Surprisingly, the angular dependent ΔR reveals obvious high frequency oscillations. By applying fast Fourier transform (FFT) analysis on the oscillations, four period can be identified as

the oscillation period of ΔR as shown in Supplementary Figure 2c, in which the first period T_1 denotes a peak at 7.8°, and the following three harmonics periods T_2, T_3 and T_4 are also visible as 15.3°, 23.3°, and 30.7°, respectively, indicating a set of high order period as $T_n = nT_1$. Therefore, the 7.8° oscillation of the angular-dependent in-plane magnetoresistivity is accordance with the twist angle of the GS. The findings presented in our article specifically pertain to GS with a large twist angle. It is worth noting that GS with a small twist angle exhibits numerous peculiar properties, and these are discussed in detail in our related work[1].

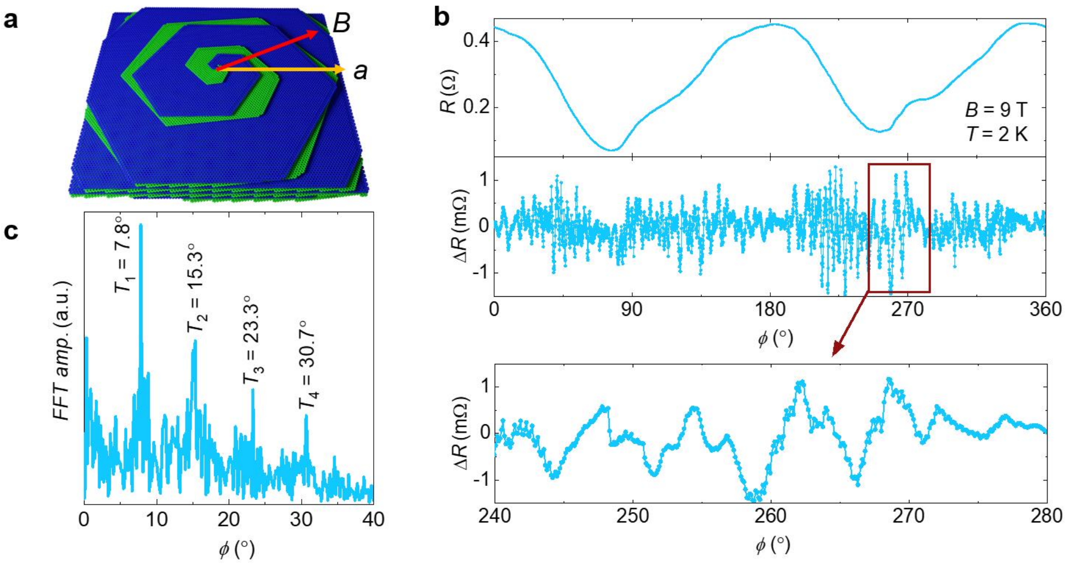

Supplementary Figure 2 Angle dependent in-plane magnetoresistance. (a) A schematic illustration of the measurement configuration. The electric current is applied within the *ab*-plane of the single crystal, and the applied magnetic field is rotated within the *ab*-plane as well. (b) Angular dependence of magnetoresistance measured at 9 T demonstrates a major two-fold symmetry owing to the Lorentz force effect from $\mathbf{B}\times\mathbf{I}$. The periodic resistance ΔR is also given by subtracting the two-fold background, and such periodic oscillation can be well identified in the enlarged view in bottom. (c) FFT spectra for the periodic resistance ΔR. Peaks of 7.8°, 15.3°, 23.3°, and 30.7° are corresponding to the 1st, 2nd, 3rd and 4th harmonics of oscillations, which are well consistent with the result of AFM and TEM.

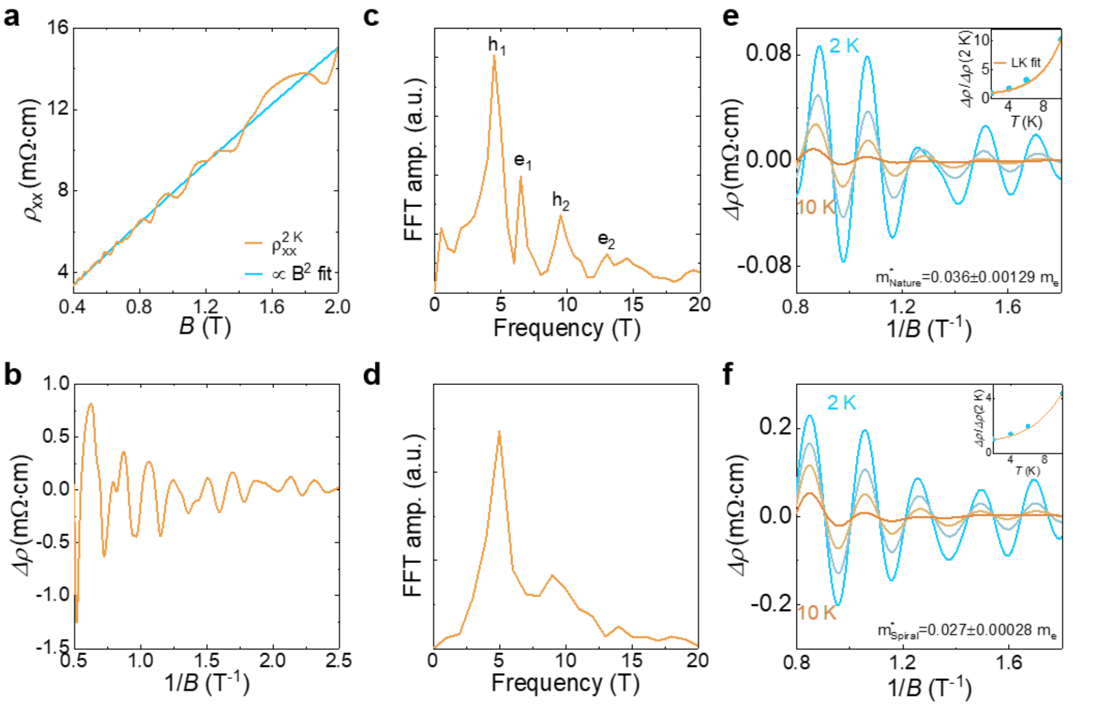

Supplementary Figure 3 Quantum oscillation. (a) ρ_{xx}(B) GS data at 2 K shows SdH oscillations. The blue line has shown the B^2 fitting which can be described as a magnetoresistance background. (b) SdH oscillations after subtracting the background from the 2 K ρ_{xx} measurements. (c) Peaks $e_{1,2}$, $h_{1,2}$ correspond to the 1st and 2nd harmonics of oscillations from electrons and holes. (d) In order to distinguish the signals from electrons and holes, a low-pass filter has been added when subtracting the background. The FFT result of filtered SdH oscillations have been shown in (d). (e, f) SdH oscillations in temperatures from 2 to 10 K. Inset shows the temperature dependence of the relative amplitude of $\Delta\rho$ for the SdH oscillation at $1/B = 0.847$ T^{-1}. The solid line is a fit to the Lifshitz–Kosevich formula: $R_T = \frac{\alpha T m^*}{m_e B} / \sinh\left(\frac{\alpha T m^*}{m_e B}\right)$, where R_T is defined as thermal damping factor, $\alpha = 2\pi^2 k_B m_e / e\hbar \approx 14.69$ T/K, k_B is Boltzmann constant, m_e is the bare mass of the electron. The result of LK fitting shows the hole effective mass of GS ($m^*_{Spiral} = 0.027 \pm 0.00028 m_e$) is smaller than NG ($m^*_{Nature} = 0.036 \pm 0.00129 m_e$).

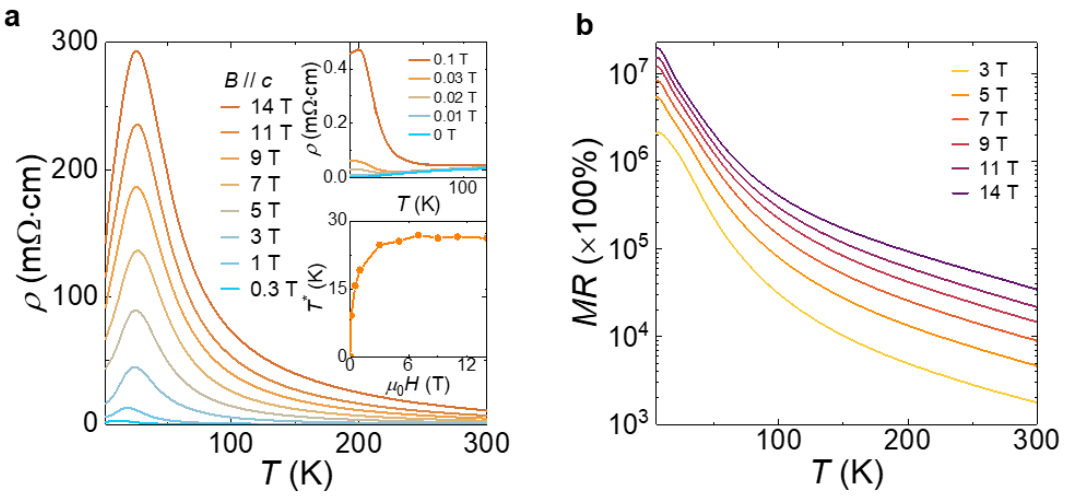

Supplementary Figure 4 Anomalous metal-insulator transition in twisted graphene spiral. (a)The temperature dependent resistivity of GS at different magnetic field from 0 to 14 T. An unconventional transition from metal to isolator appears when an infinite external magnetic field has been applied. Inset shows the magnetic field dependence of transition temperature T^{*}. (b) The temperature dependent MR at magnetic field ranging from 3T to 14 T, exhibiting an extremely large MR even at 300 K. The magnitude of MR reaches 3.4×10^{4} % at 300 K, 14 T, and exceed 10^{3} % at 300 K, 3 T.

In relatively low temperatures, MR of the twisted GS reveals obvious SdH oscillations as shown in Figure 3b. Thus, we studied the magnetoresistivity (ρ) comprehensively at 2 K under magnetic fields applied in from directions from out-of-plane to in-plane as shown in Supplementary Figure 5a. Here, the electric current was applied within the *ab*-plane, and the magnetic field was applied perpendicular to the electric current and rotated out of the *ab*-plane. θ refers to the angle between the direction of the magnetic field and the *c*-axis, as shown inset of Supplementary Figure 5a. When the field is applied away from *c*-axis, the magnetoresistivity is strongly suppressed from $\rho_{9\,\mathrm{T}}^{\theta=0^\circ}$ = 122 m$\Omega\cdot$cm to $\rho_{9\,\mathrm{T}}^{\theta=90^\circ}$ = 1.99 m$\Omega\cdot$cm, indicating the highly anisotropic nature of the carrier transport in the twisted GS. The splitting of the first oscillations (n = 1) of electron and hole can be clearly observed at $B > 3$ T (arrows in Supplementary Figure 5a), which originate from the conduction-electron factor *g*-shift in the Fermi energy under magnetic field [2-4]. It should be noted that only the perpendicular component of the magnetic field affects the band splitting due to a quasi-2D Fermi surface. Due to the weak desperation along k_z direction, one can hardly distinguish between quasi-2D behavior and 2D behavior through SdH oscillations.

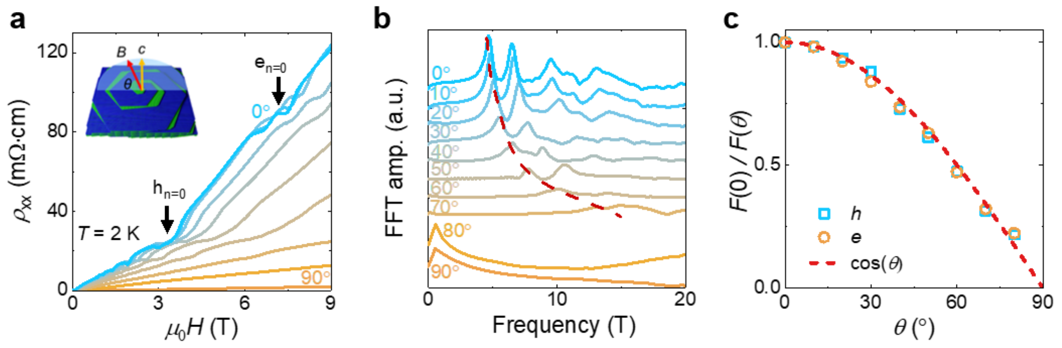

Supplementary Figure 5 Quasi-2D Fermi surface. (a) Magnetoresistivity for GS under magnetic field applied from out-of-plane (θ = 0°) to in-plane (θ = 90°). Inset shows a schematic view of magnetoresistivity measurement. The electric current is applied within the in-plane of the crystal. Here, θ corresponds to the angle between the magnetic field and the *c*-axis. Despite the presence of step-like signatures, observing the quantum Hall effect in our case proves challenging, primarily due to the considerable thickness of 70 μm. Nevertheless, as a candidate of 3D quantum Hall system, it is possible to observe some signatures such as 3D quantum Hall effect, which still deserve more experimental verification. (b) FFT analysis for the SdH oscillation in (a). Peaks correspond to the oscillations from electrons and holes. (c) Normalized FFT frequency

$f(\theta = 0) / f(\theta)$ as a function of the angle for the electron and hole oscillations, both of which obey a cos(θ)-dependence (solid line).

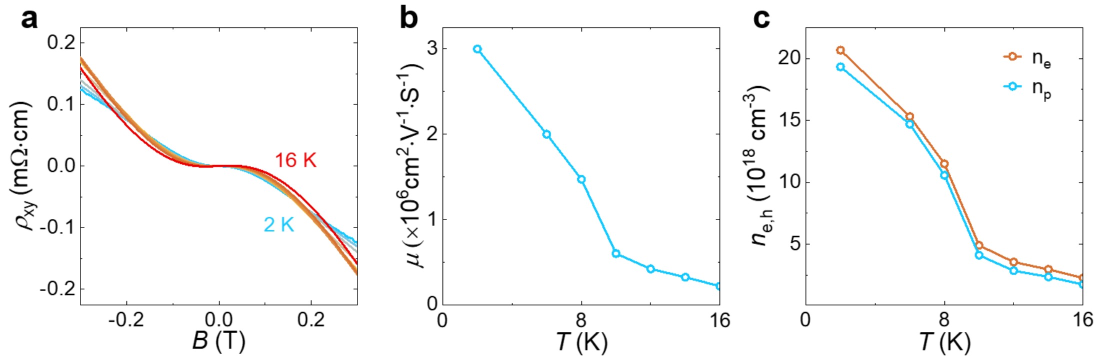

Supplementary Figure 6 High mobility and carrier concentration. (a) The Hall resistivity of GS under small magnetic fields from -0.3 T to 0.3 T, at different temperatures from 2 to 16 K. The observed nonlinear Hall curve is a characteristic of two-carrier transport, which can be described by the two-carrier model[5]: $\rho_{xy} = \frac{1}{e}\frac{(n_h\mu_h^2 - n_e\mu_e^2) + \mu_h^2\mu_e^2 B^2(n_h - n_e)}{(n_h\mu_h + n_e\mu_e)^2 + \mu_h^2\mu_e^2 B^2(n_h - n_e)^2}B$, where $n_e(n_h)$ and $\mu_e(\mu_h)$ are the carrier density and mobility of electrons (holes), respectively. An approximation ($\mu_e \approx \mu_h$) has been applied at low temperature. The temperature dependence of electron and hole mobilities from 2 to 16 K has been shown in (b). An extremely high mobility $\mu \approx 3 \times 10^6\ cm^2V^{-1}S^{-1}$ and large carrier concentration $n_{e,h} \approx 2 \times 10^{19}\ cm^{-3}$ (shown in (c)) can be observed at 2 K. The carrier concentration exhibits an anomalous increase at low temperatures, correlating with the metal-insulator transition occurring in this temperature regime. Meanwhile, the carrier concentration also follows the 3D model: $n_{3D} = k_x k_y k_z/(3\pi^2)$, where k_i represents the Fermi wave-vector along the i-direction[6]. The discrepancy between the Fermi surface and carrier concentration arises notably due to the weak dispersion along k_z.

The graphene Chemical Vapor Deposition (CVD) method

The spiral stacking structure of SP2-bonded carbon has been a subject of interest since the 1960s, as evidenced by early work. With the advent of the graphene Chemical Vapor Deposition (CVD) method, numerous research groups have demonstrated the feasibility of producing spiral graphene structures using this standard CVD process[7, 8]. Remarkably, spiral graphene structures can also be achieved by directly annealing SP2 carbon[9], underscoring the thermodynamic stability of the spiral configuration in graphene. Our recent research has further advanced this field through the development of a graphene origami-kirigami approach. This method involves processes such as wrinkling, folding, tearing, and cracking, leading to the spiral growth of graphene multilayers with controlled stacking orders. The intricacies of this graphene spiral growth process have been elaborated in our latest publication[10], providing a comprehensive understanding of this unique structural phenomenon. In current work, we more focus on the transport properties of the spiral graphene.

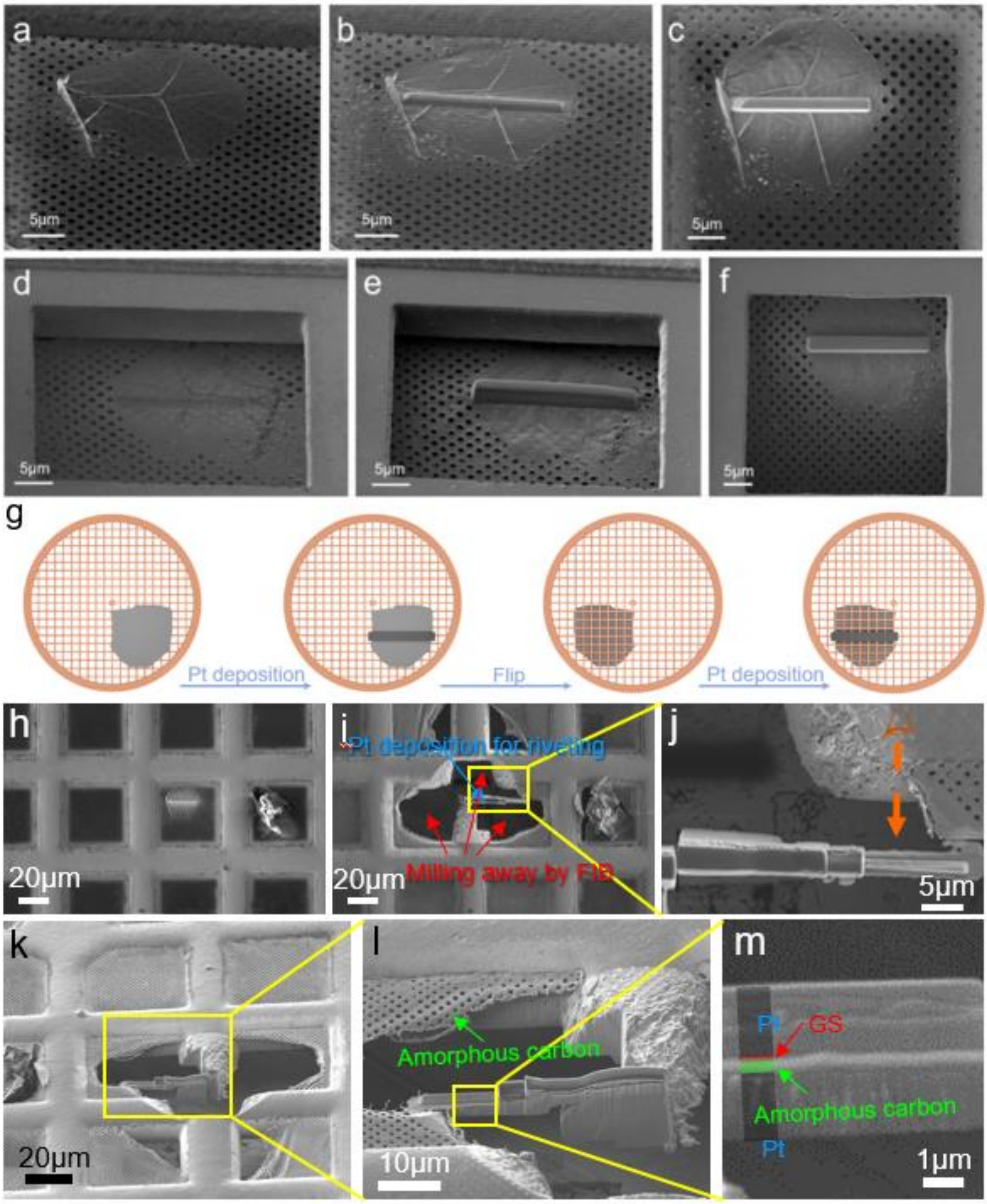

Supplementary Figure 7 The process of TEM specimen preparation for GS cross-sectional observation shown by focused ion beam. (a) SEM side view with perspective angle 45° of GS. (b) Protecting lid prepared by depositing Pt. (c) SEM top view GS after Pt deposition. (d) SEM side view with a perspective angle of 45° showing the back side of the GS. (e) Protecting lid was prepared on the back side of GS by depositing Pt. (f) SEM top view of the back side of GS after Pt deposition. (g) Schematic illustrations depicting the FIB process in a-f. (h) The zoom-out top view of GS after Pt deposition. (i) Milling on either side of the GS to get a thin wedge. (j) The zoom-in from the yellow SSwindow in (i). (k) The side view with perspective direction highlighted in (j). (l)The zoom-in from the yellow window in (k). (m) The zoom-in from the yellow window in

l.

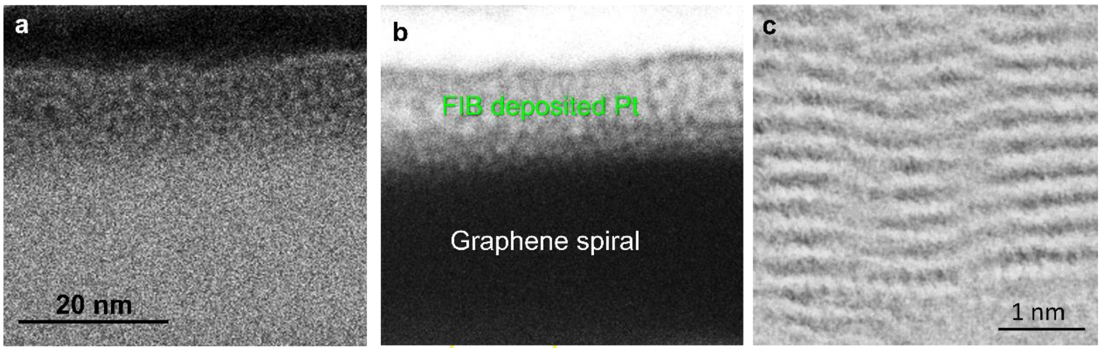

Supplementary Figure 8 GS cross-sectional STEM image. The bright-field (a) and dark-field (b) STEM images of cross-section view GS prepared via FIB processing in Figure 2. (c) The zoom-in TEM images of GS show the stacking graphene layers with staggering configuration.

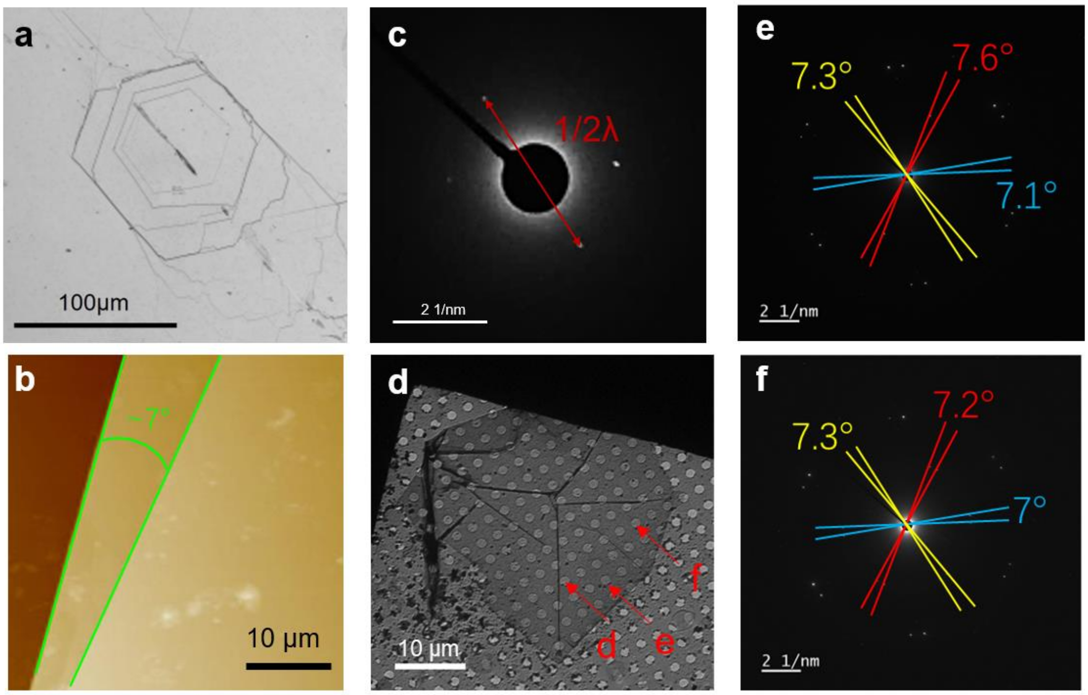

Supplementary Figure 9 The twist angle analysis using AFM and STM. (a) The CLSM image illustrates the central region of an individual GS. (b) AFM micrograph of a GS showing a fixed rotation angle of approximately $\Psi = 7.3°$ between two adjacent layers, as indicated by the marked lines. (c) Diffraction points corresponding to the moiré pattern. (d) A top-view TEM image of the GS transferred onto a TEM grid. (e-f) confirming an average twist angle of $\Psi \approx 7.3°$, which is consistent with the measurements obtained from AFM and TEM. The results in Fig. 2b come from the d region.

Normally, a twist angle between two layers of graphene can control the energy scale at which the Dirac cones of the graphene intersect in momentum space. Theoretically, the twist angle induced hexagonal moiré pattern consists of alternating AA- and AB-stacking regions, and acts as a superlattice modulation [11, 12], which will influence the 2D interface and consequently on MR. Therefore, it is essential to elucidate the effect of the twist angle on the band structure and how does it modulate the MR.

In the following note, we theoretically study the lattice deformation and electronic band structure of twisted GS. The structural relaxation is calculated utilizing Large-scale Atomic-Molecular Massively Parallel Simulation (LAMMPS)[13]. The interlayer interactions between adjacent layers are described using the Dispersion Interaction Random Phase (Drip) potential, while the intralayer potentials are described by the Adaptive Intermolecular Reactive Bond Order (AIREBO) potential[14] with a cutoff of 3 Å.. These potentials have been extensively adopted in the molecular dynamics study of twisted bilayer graphene with large twist angles. A moiré supercell with periodic boundary condition applied in both the in-plane and out-of-plane directions, serves as the initial lattice structure. The twist angles between two layers, in accordance with the commensurate condition, are ±7.34°. The steepest descent algorithm is employed in the lattice relaxation calculations, and the convergence criterion for energy is set to 10^{-8} eV. The results of the lattice relaxation are presented in Supplementary Figure 9. (a). To be specific, the arrows show the in-plane relative shift between two layers and the colorbar represent the amplitudes of the arrows. The amplitudes are approximately 10^{-4} Å, with can be considered negligible. The tendency of the lattice distortion in twisted GS is the same as that in twist bilayer graphene [15, 16]. Due to the presence of the translational symmetry along the z direction and C_{2y} symmetry, there is no out-of-plane distortion in twisted GS. Supplementary Figure 9. (b) illustrates the total energy per unit cell as a function of interlayer distance between two layers. This system exhibits the lowest total energy at an interlayer distance of 3.344 Å.

The calculation of the band structure of twisted GS is performed utilizing the tight-binding (TB) model[17, 18] based on the full relaxed structure. This model was first proposed by Moon and Koshino, and is widely adopted in the twisted graphene community. The reliability of this model has been justified by directly comparing its band structures with the density functional theory calculations for twisted bilayer graphene[19]. To be specific, The Hamiltonian is written as

$$\mathrm{H} = -\sum_{\{i,j\}} t\left(R_i - R_j\right) \left|R_i\rangle\langle R_j\right| + H.c., \qquad (1)$$

where the hopping amplitude between two $\mathrm{p_z}$orbitals at different sites is expressed as:

$$-\mathrm{t}(\mathbf{d}) = V_\sigma \left(\frac{\boldsymbol{d}\cdot\hat{\boldsymbol{z}}}{|d|}\right) + V_\pi \left[1 - \left(\frac{\boldsymbol{d}\cdot\hat{\boldsymbol{z}}}{|d|}\right)^2\right], \qquad (2)$$

where $V_\sigma = V_\sigma^0 e^{-(|d|-d_0)/\delta_0}$ and $V_\pi = V_\pi^0 e^{-(|d|-a_0)/\delta_0}$. $\mathbf{d} = (d_x, d_y, d_z)$ is the displacement vector between two sites. $d_c = 3.344$ Å is the interlayer distance. $a_0 = a/\sqrt{3}$=1.42 Å is the distance between two nearest-neighbor carbon atoms. $V_\sigma^0 = 0.48$ eV is the transfer integral between vertically located atoms on the neighboring layers and $V_\pi^0 = -2.7$ eV is that between the intralayer nearest-neighbor atoms. We set $\delta_0 = 0.184a$ so that the next-nearest intralayer coupling becomes $0.1V_\pi^0$.

The band structures calculated using the TB model at $\theta = 7.34^\circ$ for the twisted GS are showed in Fig.3 in the main text. To be specific, the band structures are calculated at several fixed values of k_z. The band width of flat bands exhibits an increase as k_z increases. The minimum band width is approximately 1.3 eV at $k_z = 0$. No indirect gap is identified as k_z increases. At $k_z = \pi / 2d_c$, the band structures resemble that of a single layer graphene, implying that the effects caused by each pair of adjacent moiré potentials are canceled. Besides, the lattice distortions result in splittings in certain energy bands.

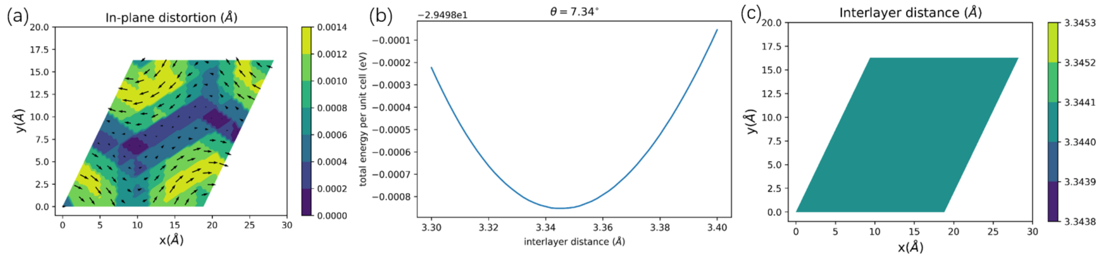

Supplementary Figure 10 The lattice relaxation pattern of GS. (a) The real space distribution of the in-plane lattice distortions of twisted GS. The arrows show the directions of the in-plane distortion field, while the color bar represents the amplitudes of the distortion field. (b) The total energy per unit cell calculated by LAMMPS, as a function of interlayer distance. (c) The real space distribution of the interlayer distance, indicating that there is almost no out-of-plane corrugations.

In previous calculation, we consider the lattice relaxation effects and the electronic properties far away from the dislocation core. Here we will justify this treatment. Firstly, we consider the initial lattice structure as a Graphite Spiral with the dislocation line located at the AA point in the moiré supercell. We consider the lattice dislocation line in the z direction, while neglecting the in-plane displacement. The lattice distortion in the z direction at position **r** (by setting the dislocation line as the origin) can be expressed as:

$$u_z(\boldsymbol{r}) = \frac{2d_0}{2\pi}\arg(r_x + i\, r_y),$$

where the function arg denotes the argument of the complex number, and d_0 is the interlayer distance between two adjacent graphene layer. The presence of dislocation line breaks the translational symmetry. Thus, we set an open boundary condition in the x-y direction and a periodic boundary condition in the z direction. We construct a 12×12 supercell for GS with twist angle $\theta = 7.34°$. The corresponding lattice constant of the supercell is 1.92 nm. We perform a lattice relaxation calculation with LAMMPS (see detailed software settings in the following reply). In Supplementary Figure 11, we present the results of the lattice relaxation calculation. We plot the distortion form an exact flat plane in the z direction as a function of distance from the dislocation line within one graphene layer. The distortion in z direction decays rapidly within 1 nm. In other words, the lattice distortion induced by the dislocation core is localized within a distance of 1~2 nm. Since the size of our sample is much larger, we neglect the lattice distortion effects induced by the dislocation core.

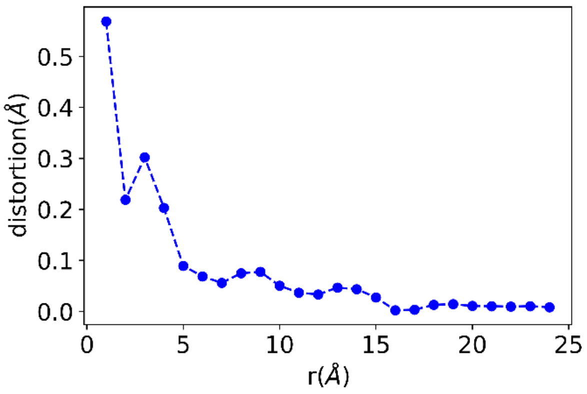

Supplementary Figure 11. The distortion in the z direction as a function of the distance from the dislocation line in one graphene layer.

Besides, in order to investigate the influence of the dislocation core on the electronic properties, we calculate the calculate the electronic band structure of GS by using the tight binding model described in the supplementary information based on the fully relaxed 12×12 supercell with dislocation line. Then, we project the energy band to the nearest neighbor atoms of the dislocation line and evaluate the spectral function of the bounded states near the dislocation core. In Supplementary Figure 12, we present the spectral function of the bounded states near the dislocation line for GS with a 12×12 supercell and twist angle $\theta = 7.34°$. The bounded states near the Fermi level are very weak. As a result, we neglect the influence of the dislocation in the calculation of electronic properties.

In order to investigate the influence of the finite lager number, in other words, finite size effects in the z direction, we have calculated the top surface states of GS with twist angle $\theta = 7.34°$ using the iterative Green's function methods, as shown in Supplementary Figure 13. We find that the surface-state spectra are basically the same the bulk ones, and there is no topological surface state. We see that the surface states and bulk states are nearly identical. As a result, we neglect the influence of the top and

bottom surface on the electronic properties.

Based on the preceding argument, it is justifiable to neglect the dislocation and consider the lattice relaxation effects both near and far away from the dislocation core in our simulation.

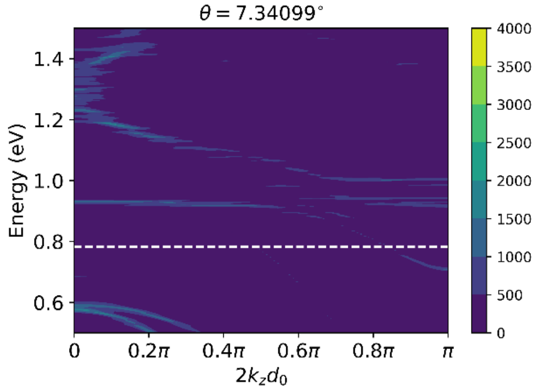

Supplementary Figure 12. The bounded states near the dislocation line based on the fully relaxed GS with a 12 × 12 supercell and twist angle θ = 7.34°. The white dashed line represents the Fermi level.

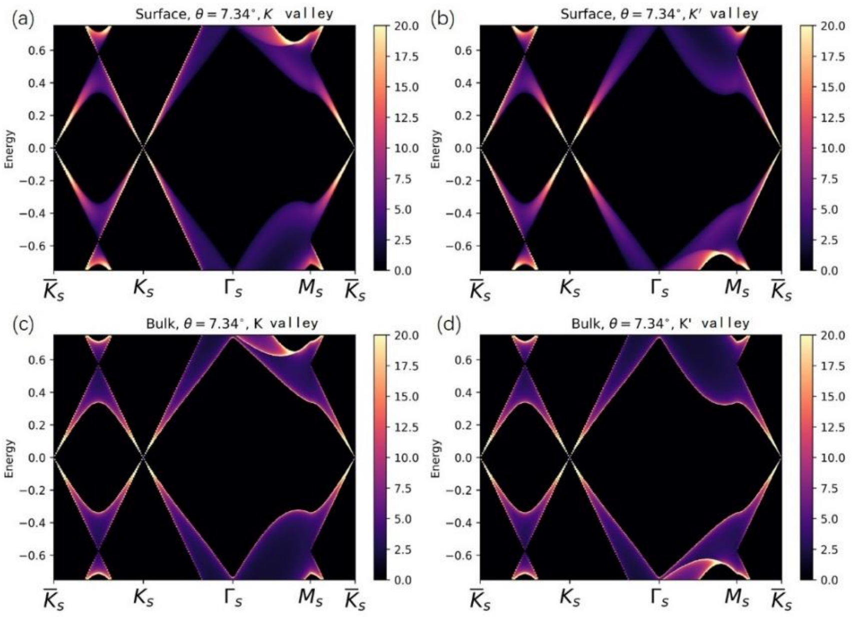

Supplementary Figure 13 Energy spectra of the surface states of alternating twisted graphite with θ = 7.34°, (a) from the K valley, and (b) from the K’ valley. Energy spectra of the bulk states of alternating twisted graphite with θ = 7.34°, (c) from the K valley, and (d) from the K’ valley.

Supplementary References

1. Lu, X., et al., Magic Momenta and Three-Dimensional Landau Levels from a Three-Dimensional Graphite Moiré Superlattice. Physical Review Letters 132(5) (2024).
2. Woollam, J.A., Spin Splitting, Fermi Energy Changes, and AnomalousgShifts in Single-Crystal and Pyrolytic Graphite. Physical Review Letters 25(12) (1970).
3. Schneider, J.M., et al., Using magnetotransport to determine the spin splitting in graphite. Physical Review B 81(19) (2010).
4. Schneider, J.M., et al., Consistent interpretation of the low-temperature magnetotransport in graphite using the Slonczewski-Weiss-McClure 3D band-structure calculations. Phys Rev Lett 102(16) (2009).
5. Smith, R.A., Semiconductors. (1964).
6. Tang, F., et al., Three-dimensional quantum Hall effect and metal–insulator transition in ZrTe5. Nature 569(7757) (2019).
7. Wang, Z.-J., et al., Formation Mechanism, Growth Kinetics, and Stability Limits of Graphene Adlayers in Metal-Catalyzed CVD Growth. Advanced Materials Interface 5(14) (2018).
8. Tay, R.Y., et al., Concentric and spiral few-layer graphene: growth driven by interfacial nucleation vs screw dislocation. Chemistry of Material 30(19) (2018).
9. Sun, Y., et al., Structural dislocations in anthracite. The Journal of Physical Chemistry Letters 2(20) (2011).
10. Wang, Z.-J., et al., Conversion of chirality to twisting via sequential one-dimensional and two-dimensional growth of graphene spirals. Nature Materials 23(3) (2024).
11. Bistritzer, R. and A.H. MacDonald, Moire bands in twisted double-layer graphene. Proceedings of the National Academy of Science 108(30) (2011).
12. Fang, S. and E. Kaxiras, Electronic structure theory of weakly interacting bilayers. Physical Review B 93(23) (2016).
13. Thompson, A.P., et al., LAMMPS-a flexible simulation tool for particle-based materials modeling at the atomic, meso, and continuum scales. Computer Physics Communication 271 (2022).
14. Leconte, N., et al., Relaxation effects in twisted bilayer graphene: A multiscale approach. Physical Review B 106(11) (2022).
15. Angeli, M., et al., Emergent D_6 symmetry in fully relaxed magic-angle twisted bilayer graphene. Physical Review B 98(23) (2018).
16. Nam, N.N.T. and M. Koshino, Lattice relaxation and energy band modulation in twisted bilayer graphene. Physical Review B 96(7) (2017).
17. Slater, J.C. and G.F. Koster, Simplified LCAO Method for the Periodic Potential Problem. Physical Review 94(6) (1954).
18. Moon, P. and M. Koshino, Optical absorption in twisted bilayer graphene. Physical Review B 87(20) (2013).
19. Zhang, T., et al., O(N) ab initio calculation scheme for large-scale moiré

structures. Physical Review B 105(12) (2022).
